# Gene Expression in *Cucurbita* spp. Root and Crown during *Phytophthora capsici* Infection

**DOI:** 10.3390/plants10122718

**Published:** 2021-12-10

**Authors:** Alejandro Ayala-Doñas, Pedro Gómez, Miguel de Cara-García

**Affiliations:** Andalusian Institute of Agricultural and Fisheries Research and Training, IFAPA La Mojonera, Autovía del Mediterráneo, 420, Paraje San Nicolás, La Mojonera, 04745 Almería, Spain; alejandro.ayala@juntadeandalucia.es (A.A.-D.); pedro.gomez.j@juntadeandalucia.es (P.G.)

**Keywords:** *Cucurbita pepo*, *Cucurbita moschata*, defensin, tolerance, susceptibility, squash

## Abstract

*Phytophtora capsici* causes major diseases in cucurbit crops worldwide. In this study, we inoculated this pathogen into *Cucurbita pepo* subsp. *pepo* susceptible MUCU-16 and *C. moschata* tolerant M63. The gene expression of plant pathogenesis-related proteins chitinase (*CpChiIV*), lignin-forming peroxidase (*CpLPOX*), and defensin (*CpDEF*) and hormone-related enzymes salicylic acid (*CpPAL*) and ethylene (*CpACO*) was analyzed for two weeks post-inoculation in root and crown tissues. Differentially expressed genes were found between genotypes, tissues, days post-inoculation, and inoculated/non-inoculated samples. After inoculation, *CpPAL* and *CpChiIV* (crown) were downregulated in MUCU-16, while *CpLPOX* and *CpDEF* were upregulated in M63. In inoculated samples, higher expression changes were presented on days 10–14 than on day 3 for *CpACO*, *CpLPOX*, and *CpDEF* genes. Overexpression was higher for *CpDEF* compared to the other tested genes, indicating good suitability as a marker of biotic stress. The overexpression of *CpDEF* was higher in crown than in roots for both inoculated genotypes. The basal expression of *CpPAL* and *CpDEF* was higher in MUCU-16, but after inoculation, *CpPAL* and *CpDEF* gene expression were higher in M63. These changes suggest an association between *CpDEF* upregulation and tolerance, and between *CpPAL* downregulation and susceptibility.

## 1. Introduction

Squashes (*Cucurbita* spp.) are a heterogeneous genus in terms of cultivable species and morphotypes. They are distributed worldwide and some have economic significance, such as gourds and pumpkins (*Cucurbita moschata* and *Cucurbita maxima*, among others) and zucchini (*Cucurbita pepo* subsp. *pepo*). Their production can be severely affected by soil-borne pathogens, such as the oomycete *Phytophthora capsici*, which causes foliar blight and root, crown (basal stem), and fruit rot in a wide range of cucurbit species [1]. This pathogen, when present in the soil, causes damage to the crown that leads to stem girdling and plant wilting due to the inability to transport nutrients. *P. capsici* commonly appears in over-irrigated fields and can survive in plant residues or in the soil for long periods as oospores, which allows the fungi to overcome extreme temperatures or desiccation [1,2]. Therefore, current integrated management relies on soil water management, chemical treatment of soil, seeds, and plants, bio/solarization, or crop rotation [2,3].

Plant resistance has been proven as an efficient method to control fungal diseases; however, there is a lack of commercially available squash varieties with resistance to *P. capsici*. Reduced crown damage caused by *P. capsici* has been found in *Cucurbita lundelliana* and *C. okeechobeensis* wild species [4], *C. pepo* [5], and *C. moschata* [6]. Resistance to *P. capsici* derived from *Cucurbita lundelliana* and *C. okeechobeensis* has been introgressed into *C. moschata*, and its segregation suggests that this trait is controlled by three dominant genes [7]. Furthermore, in a screening of resistance to *P. capsici* in *C. pepo* species, it was found that 16 accessions were less damaged by the pathogen [5]. Backcrossing and phenotypic segregation tests also indicated that the resistance to *P. capsici* was also controlled by three dominant genes [8]. However, there is still no cultivar resistant to this disease in the highly susceptible *Cucurbita pepo* subsp. *pepo* genotype, despite its being one of the main cultivable crops in the *Cucurbitaceae* family.

The molecular interaction between cucurbits and soil-borne pathogenic fungi has been studied from various perspectives, and some components involved have been described. However, most studies have used *Fusarium oxysporum* and other crops in the *Cucumis* genus [9,10], while molecular studies of *Phytophthora capsici* have used horticultural crops such as pepper [11,12]. Many of the plant defensive tools against soil-borne pathogenic fungi are enzymes that act directly on pathogens, such as chitinases, 1,3-glucanases/glucosidases, and peroxidases [13,14,15]; are involved in the production of defense structures such as lignin [16], tylose [17], and suberin [18]; are antimicrobial molecules (phytoalexins) [19]; or are oligopeptides (defensins) [14]. 

The chitinases, whose expression is enhanced by numerous biotic and abiotic factors, hydrolyze the N-acetylglucosamine polymer chitin. Chitin is a structural component of the cell wall of many phytopathogenic fungi, so its degradation has been related to fungal resistance [20]. Another highly studied family is the peroxidases, of which there are several types. The ones that are more closely related to plant defense are involved in reactive oxygen species production or in processes such as suberization or lignification. Lignin-forming peroxidases act at the final stages of lignin biosynthesis, forming part of the cell wall of many plant cells, especially the cells of the xylem conduct, which pathogenic fungi use to penetrate plants [21]. Plant defensins are highly stable, cysteine-rich oligopeptides that are found in a wide variety of crops and have conserved structures depending on the cysteine position, which categorizes them into different families [22]. These defensins are part of the plant’s innate immune system and have important antifungal activity, and many studies have linked their overexpression to enhanced disease control [23,24]. All of these pathogenesis-related components are closely regulated by phytohormones such as salicylic acid, jasmonic acid, or ethylene, which are also involved in the plant’s defensive response [9]. Generally, the salicylic acid route is related to plant pathogen tolerance, whereas jasmonic acid, ethylene, abscisic acid, and auxin have complex effects. These hormone signaling pathways are often targeted by pathogens, which can interfere with them [25].

The aim of this work was to describe the expression of some genes that may be part of the *Cucurbita* spp. response to *P. capsici* inoculation. For this purpose, we analyzed the gene expression of the susceptible cultivar MUCU-16 (*C. pepo* subsp. *pepo*) and the tolerant cultivar M64 (*C. moschata*) in root and crown tissues for the first two weeks post-inoculation. Three pathogenesis-related proteins—EP3 endochitinase (*CpChitIV*), lignin-forming peroxidase (*CpLPOX*), and J1-2 defensin (*CpDEF*)—were selected for the analysis. In addition, two enzymes related to hormone biosynthesis, phenylalanine ammonia-lyase (*CpPAL*), which favors the production of salicylic acid precursors [26], and 1-aminocyclopropane-1-carboxylate oxidase (*CpACO*, ethylene), which participates in the last step of ethylene biosynthesis, converting the 1-aminocyclopropane-1-carboxylic acid into ethylene [27].

## 2. Results

### 2.1. Phytophthora capsici Disease Evolution in Cucurbita spp.

**Figure 1:**
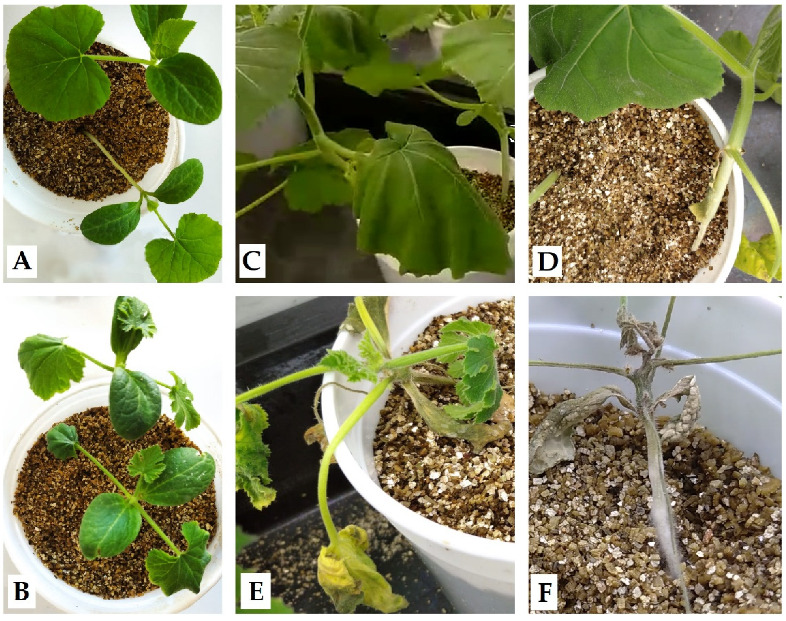
*Phytophthora capsici* symptomatology in *Cucurbita* spp. (**A**) M63 and (**B**) MUCU-16 3 days post-inoculation; only MUCU-16 showed damage (leaf decay). After 14 days post-inoculation, some M63 plants showed (**C**) leaf decay or (**D**) soaked/girdling crown, while MUCU-16 presented (**E**) wilt/chlorosis and (**F**) death.

The tolerant *C. moschata* M63 population showed a lower disease severity index (DSI) than the *Cucurbita pepo* subsp. *pepo* susceptible MUCU-16 (*p* = 0.0012; Table 1), although both species were damaged by *P. capsici*. The symptoms in affected plants were similar. Initially, the cucurbits showed leaf decay and the pathogen progressively damaged the crown, causing girdling and soaking, which led to plant wilting (Figure 1). The symptomatology was on set on day 3 for the susceptible and day 10 for the tolerant species.

### 2.2. Normalized Relative Quantity

**Figure 2:**
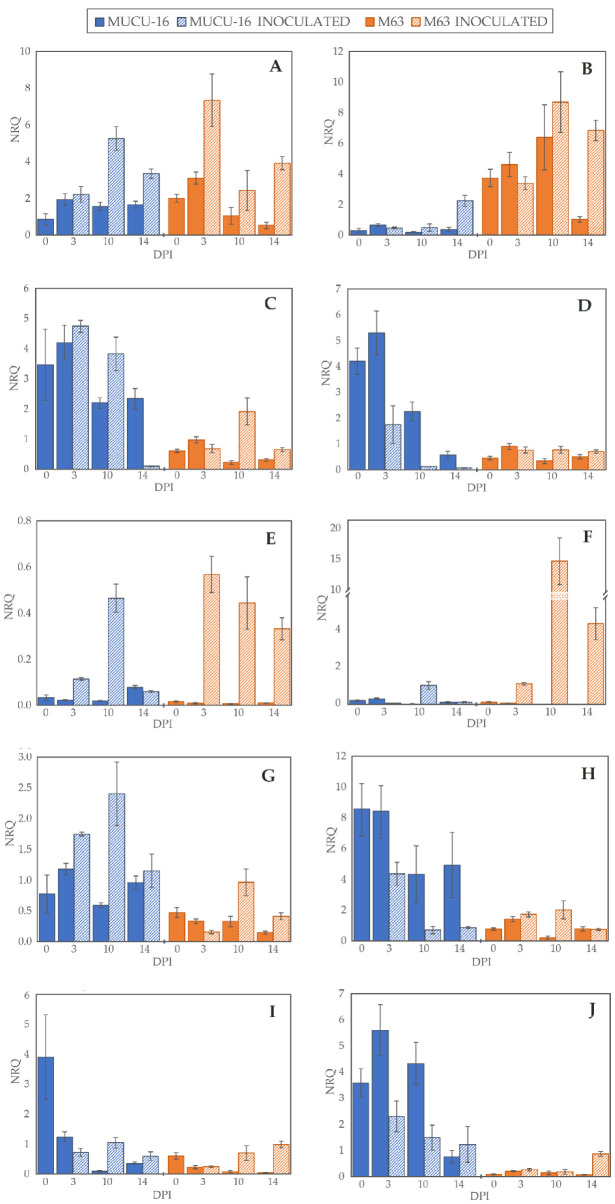
Normalized relative quantity (NRQ) of selected genes. NRQ (*Y*-axis) for 0, 3, 10, and 14 days post-inoculation (*X*-axis) of genotypes MUCU-16 (blue) and M63 (orange) and inoculated (striped bars) and non-inoculated (solid bars) samples: (**A**,**B**) 1-aminocyclopropane-1-carboxylate oxidase (*CpACO*) expression in root and crown, respectively; (**C**,**D**) phenylalanine ammonia-lyase (*CpPAL*) expression in root and crown; (**E**,**F**) defensin (*CpDEF*) expression in root and crown; (**G**,**H**) endochitinase EP3 (*CpChiIV*) expression in root and crown; (**I**,**J**) lignin-forming peroxidase (*CpLPOX*) expression in root and crown. Data were relativized with UBI and EF1a as reference genes [29]. Error bars represent standard error from 3 replicates.

Gene expression changes were observed between genotypes, tissues, inoculated/non-inoculated samples, and time post-inoculation (Figure 2, Table 2, Table 3 and Table 4).

On day 3 post-inoculation, fewer changes between inoculated and non-inoculated samples occurred than on days 10–14 (*p* = 0.004, *n* = 120) when the data of all genes for the same day were pooled. After inoculation, *CpLPOX* and *CpDEF* were upregulated only in M63, while no gene was significantly upregulated in MUCU-16 (Table 2). *CpACO* gene was upregulated on days 10–14 post-inoculation, suggesting a late plant response through this gene (Table 2). In inoculated samples, *CpDEF* was significantly upregulated in both crown and root, while *CpACO* was only in root (Table 2).

Pooling all the samples, there were no significant differences between tissues, with the exception of *CpChiIV*, which was more expressed in crown than in root (Table 3). However, for the inoculated samples, this difference in *CpChiIV* expression disappeared, suggesting an effect of the tissue for this gene (Table 3). In addition, in MUCU-16, *CpLPOX* and *CpACO* were significantly more expressed in crown and root, respectively (Table 3).

Regardless of the origin of the samples (e.g., day sampling, inoculated/non-inoculated, tissue), the highest differences were found between genotypes, especially in non-inoculated samples (Table 4). All genes tested showed more basal expression in MUCU-16 than in M63, except for *CpACO*, which was more expressed in M63 (Figure 2, Table 4). These differences were significant in the crown for *CpACO*, *CpChiIV*, and *CpLPOX* and in the roots for *CpChiIV* and *CpPAL* (Table 4).

Due to the influences that each analyzed factor (treatment, tissues, genotype and days post-treatment) could have on the interpretation of more than one factor simultaneously, they were relativized by their levels (inoculated/non-inoculated, crown/root, M63/MUCU-16) and reinterpreted in the following sections.

### 2.3. Differentially Expressed Genes between Inoculated and Non-Inoculated Plants

**Figure 3:**
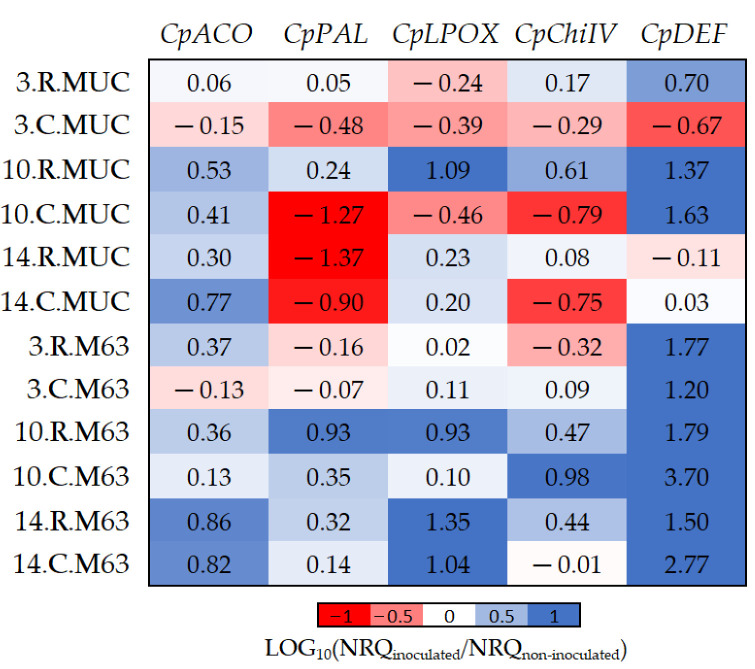
The log_10_ fold changes between inoculated and non-inoculated samples. NRQ was transformed with LOG_10_(NRQ_inoculated_/NRQ_non-inoculated_). Sample nomenclature: days post-inoculation (3, 10, and 14); root (R) or crown (C); M63 or MUCU-16 (MUC). Each unit represents a 10-fold change in expression of inoculated compared to non-inoculated samples; positive values (blue) mean higher expression in inoculated samples and negative values (red) mean higher expression in non-inoculated samples. *CpACO*, 1-aminocyclopropane-1-carboxylate oxidase; *CpPAL*, phenylalanine ammonia-lyase; *CpLPOX*, lignin-forming peroxidase; *CpChiIV*, endochitinase EP3; *CpDEF*, defensin.

To avoid the influence of the background gene expression of tissues and genotypes (Table 3 and Table 4), data were relativized comparing inoculated versus non-inoculated samples (Figure 3). The results in Figure 3 show how the tissues (*CpChiIV*) and genotypes (*CpPAL* and *CpDEF*) selected could affect the differential expression between inoculated and non-inoculated samples.

While *CpDEF* was upregulated in inoculated plants (Figure 3, Table 2), the upregulation was significantly higher in M63 (Table 5). The *CpPAL* gene was significantly more expressed in M63 (especially in crown) due to the downregulation of MUCU-16 after inoculation (Table 5, Figure 3). The lack of *CpDEF* upregulation on day 14 post-inoculation in both tissues for MUCU-16 (Figure 3) was also remarkable. 

**Figure 4:**
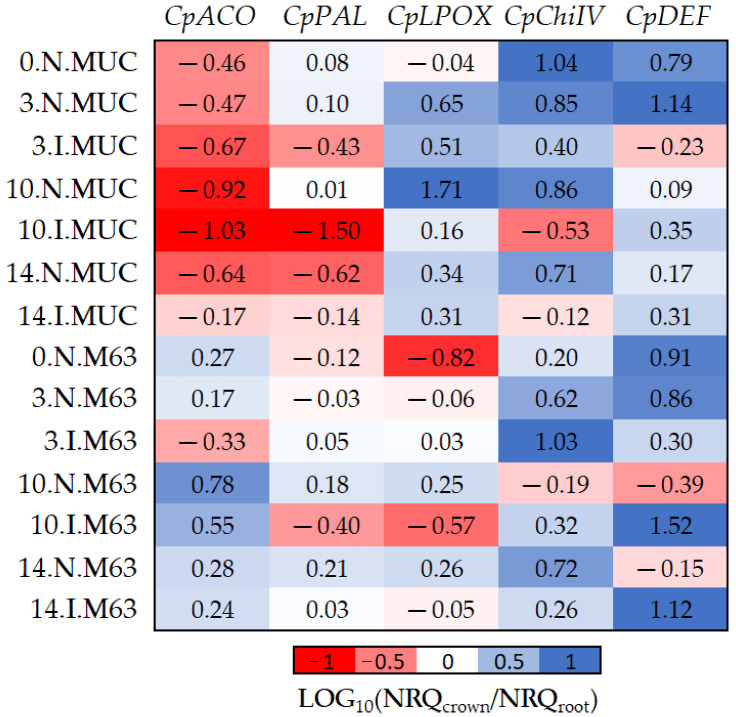
The log_10_ fold changes between crown and root samples. NRQ was transformed with LOG_10_(NRQ_crown_/NRQ_root_). Sample nomenclature: days post-inoculation (0, 3, 10, and 14); inoculated (I) or non-inoculated (N); M63 or MUCU-16 (MUC). Each unit represents 10-fold change in expression of crown compared to root samples; positive values (blue) mean higher expression in crown and negative values (red) mean higher expression in roots. *CpACO*, 1-aminocyclopropane-1-carboxylate oxidase; *CpPAL*, phenylalanine ammonia-lyase; *CpLPOX*, lignin-forming peroxidase; *CpChiIV*, endochitinase EP3; *CpDEF*, defensin.

To avoid the influence of the inoculation and genotype background on gene expression (Table 2 and Table 4), data were relativized comparing crown versus root tissues (Figure 4). The results in Figure 4 show how the inoculation (*CpDEF* and *CpChiIV)* and genotype (*CpACO* and *CpLPOX*) affect the tissue expression pattern.

The *CpDEF* gene was upregulated in both the crown and root tissues in inoculated samples (Table 2), but the upregulation was higher in the crown (Figure 4, Table 6) on days 10–14. In addition, it was only in MUCU-16 that the *CpChiIV* gene significantly changed its tissue expression and was less expressed in crown than in root in inoculated samples (Figure 4, Table 6). These tissue expression changes due to inoculation were significant on days 10–14 (Figure 4, Table 6), because higher expression changes due to inoculation were presented on these days over that of day 3 (Table 2). 

Furthermore, significant differences in *CpACO* and *CpLPOX* tissue expression patterns were also observed between genotypes (Figure 4). Pooling inoculated and non-inoculated samples, MUCU-16 *CpACO* (*p* < 0.001, *n* = 12) and *CpLPOX* (*p* = 0.035, *n* = 12) were more associated with root and crown, respectively, compared to M63 (Figure 4).

### 2.4. Differentially Expressed Genes between Tolerant and Susceptible Genotypes

**Figure 5:**
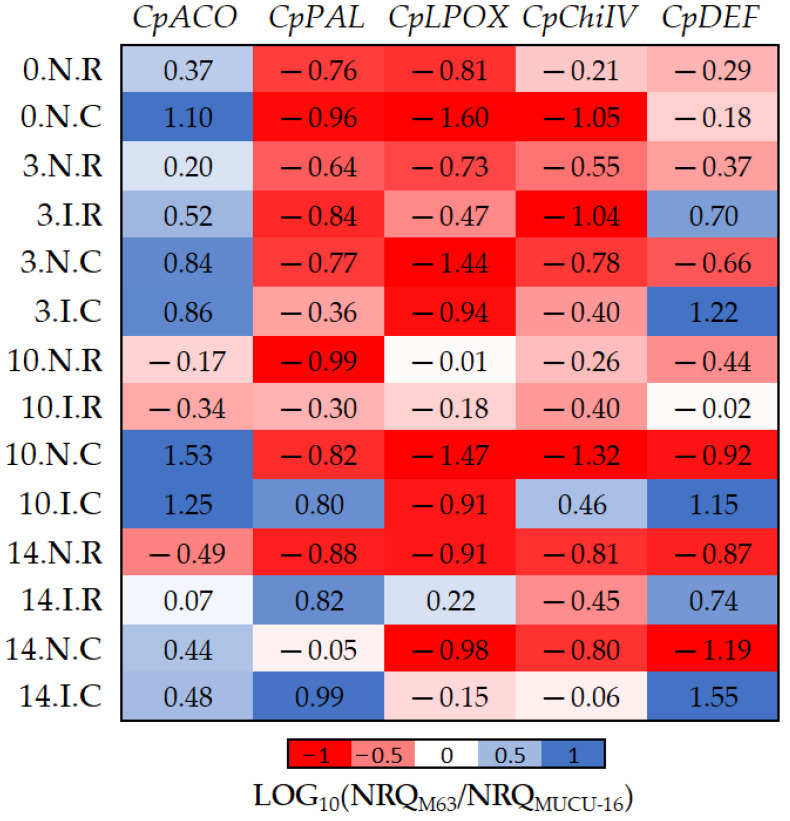
The log_10_ fold changes between M63 and MUCU-16 samples. NRQ was transformed with LOG_10_(NRQ_M63_/NRQ_MUCU-16_). Sample nomenclature: days post-inoculation (0, 3, 10, and 14); inoculated (I) or non-inoculated (N); root (R) or crown (C). Each unit represents 10-fold change in expression of the M63 compared to MUCU-16 samples; positive values (blue) mean higher expression in M63 and negative values (red) mean higher expression in MUCU-16. *CpACO*, 1-aminocyclopropane-1-carboxylate oxidase; *CpPAL*, phenylalanine ammonia-lyase; *CpLPOX*, lignin-forming peroxidase; *CpChiIV*, endochitinase EP3; *CpDEF*, defensin.

To avoid the influence of the inoculation and tissue background on gene expression (Table 2 and Table 3), data were relativized comparing M63 versus MUCU-16 genotypes (Figure 5). The results in Figure 5 show how the inoculation (*CpDEF*, *CpPAL, CpChiIV)* and tissue (*CpACO* and *CpLPOX*) affect the genotype differential expression.

Basal (non-inoculated) expression of all genes was higher in susceptible plants, with the exception of *CpACO* (Table 4, Figure 5). However, inoculation led to higher expression of *CpDEF*, *CpChiIV*, and *CpPAL* genes in M63. The *CpChiIV* upregulation was only observed in crown and *CpPAL* only on days 10–14, but *CpDEF* was upregulated in all circumstances (Figure 5, Table 7). These differences between inoculated and non-inoculated samples were especially observable between days 10 and 14 (Figure 5).

Furthermore, significant differences in *CpACO* (*p* = 0.002, *n* = 12) and *CpLPOX* (*p* = 0.036, *n* = 12) expression were also observed between the tissues of each genotype (Figure 5). Pooling inoculated and non-inoculated samples, the differential expression among genotypes for *CpACO* differed between crown and roots, because it was more expressed in M63 crown. On the other hand, differential expression among genotypes for *CpLPOX* also differed between tissues, because this gene was more expressed in MUCU-16 crown (Figure 5).

## 3. Discussion

To our knowledge, this is the first gene expression study of *Cucurbita* spp. inoculated with *Phytophthora capsici.* We studied the expression of genes that other authors have described as being involved in plant responses to biotic stress and we compared the two main tissues damaged by the pathogen, root and crown, in two species, *Cucurbita pepo* subsp. *pepo* susceptible MUCU-16 and *Cucurbita moschata* tolerant M63, during disease development for two weeks. 

Basal expression (non-inoculated) differences between species (Table 4) can be explained by the qPCR reaction efficiency. However, we cannot discard the influence of plant development differences such as biomass or architecture. Due to the varied nature of the samples, the influences of inoculation, tissue, and species were corrected through data relativization (Figure 3, Figure 4 and Figure 5). This relativization and subsequent logarithmic transformation allowed new and more reliable information to be found among samples. 

The study of gene expression to understand how plants and pathogens interact and react molecularly to each other has already been applied to some horticultural crops. Priming of the plant immune system occurs through resistance genes, as in pathogenesis-related proteins *CaPBR1*, *CaPO1*, and *CaDEF1* for *Phytophthora capsici* in pepper (*Capsicum annuum*) [30]. Likewise, changes in transcript levels have also been observed for *Fusarium oxysporum* f. sp. *niveum* (FON) during infection of watermelon, whose defensin-like genes *ClPDF2.1* and *ClPDF2.4*, phenylalanine ammonia lyase, chitinase, and ascorbate peroxidase were significantly induced in roots inoculated with FON race 1, while polyphenol oxidase did not show a significant response to FON 1 infection [14]. 

In plants, ethylene biosynthesis and downstream signaling occur through a conserved pathway that leads to a cascade of transcription factors that regulate ethylene-mediated responses, including various plant defense mechanisms against pathogens [31]. Wang et al. (2015) [12] showed that in pepper roots infected with *P. capsici*, there was upregulation of genes related to ethylene and jasmonic acid. Similarly, a melon with *P. capsici* resistance (ZQK9) showed upregulation of the ACO gene, while for susceptible plants (E31) this gene was downregulated in the roots at 3 and 5 DPI [10]. Our results indicate that the main upregulation of *CpACO* occurred once the disease had developed on days 10–14 (Table 2).

Plant chitinases are known as pathogenesis-related proteins induced in response to attacks by phytopathogens, elicitors, or growth regulators [32]. However, our results did not show an evident relationship between ethylene (*CpACO*) biosynthesis and chitinase (*CpChiIV*) expression. The *CpChiIV* gene encodes a class IV chitinase that targets the chitin of the true fungal cell wall. *Phytophthora capsici*, as an oomycete, does not possess chitin, although increased expression of a type IV chitinase has been observed in response to *P. capsici* in leaves of pepper plants [11]. Our results point out that in inoculated samples, the chitinase was more downregulated in the susceptible crown, while its expression was maintained in the tolerant plant (Table 7). It is important to highlight the relevance of the sample used for each analysis, since the study by Liu et al. (2017) [11] used pepper leaf tissue, because *CpChiIV* was downregulated in the MUCU-16 crown (Table 7), but not in its roots. 

The activation of the salicylic acid-dependent pathway leads to the overexpression of genes that encode pathogenesis-related proteins, such as defensins and peroxidases [30]. We have seen that in inoculated samples, the downregulation of *CpPAL* in susceptible plants was similar to the *CpChiIV* only in the crown. No other patterns were observed, so their direct relationship was unclear. In *P. capsici*-inoculated melon roots, the PAL gene was upregulated in a resistant cultivar (ZQK9) and in susceptible plants (E31), although PAL expression was higher in the resistant melon [10]. In our work, *CpPAL* was downregulated in MUCU-16 during *P. capsici* infection compared to M63 (Table 5). This observation corresponds with the results of Li et al. (2019) [33], who pointed out that *P. capsici* suppresses the plant’s defensive response by targeting salicylic acid signaling. Plants that are defective in salicylic acid synthesis/accumulation always exhibit enhanced susceptibility [33,34], and we have seen a connection between susceptibility and downregulation of *CpPAL*. The situation was different for *CpPAL* and *CpChiIV*, since *CpChiIV* was only downregulated in the MUCU-16 crown in inoculated samples (Table 7), the expression levels were not significantly different from tolerant plants (Table 5), so *CpChiIV* downregulation did not seem as closely related to susceptibility as *CpPAL* downregulation.

**Figure A1:**
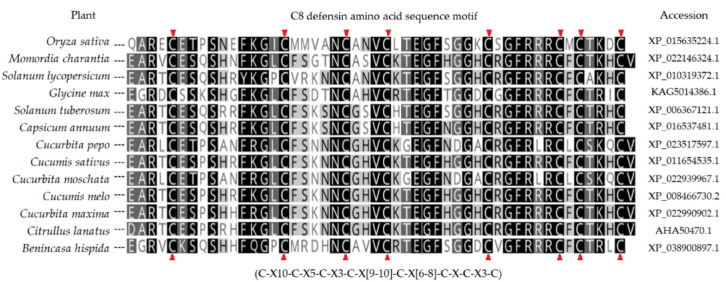
C8 defensin amino acid sequence in cucurbits and other crops.

Analogous to our results (Figure 3, Table 5), for defensin-like genes, overexpression in *P. capsici*-inoculated roots was higher in tolerant melon (ZQK9) than in susceptible melon (E31) [10]. The defensin that was analyzed corresponds to the group with C8 structure [22], which has eight cysteines in a specific position, and whose motif is found across multiple animals, plants, and fungi. This type of defensin is found in several cucurbit plants as well as in other important crop species (Appendix A, Figure A1), and it could be a gene whose overexpression indicates infectious disease stress in all of them. In addition, as other studies [10,14] have also indicated, increased expression could favor disease tolerance, making it a gene of great interest for many cultivable crops. 

Lignin accumulation is a general defense mechanism against fungal penetration; higher lignin concentration around the appressoria has been observed in resistant crops [16]. Furthermore, histochemical staining of *P. capsici*-inoculated roots showed that the epidermal cells of a susceptible melon (E31) were disintegrated, while the cellular structure of the resistant plant (ZQK9) showed no important changes [10]. A gene encoding a type of lignin-forming peroxidase was upregulated in *Phytophthora capsici*-inoculated samples of *Capsicum annuum* [12] and in roots of the resistant *Cucumis melo* (ZQK9) [10]. Both results were similar to ours for the *CpLPOX* gene, although we have seen that this response can also occur in the crown.

To compare results with other studies, it must be taken into account that a resistant cultivar may not give the same results as a tolerant one (like M63) whose tissues are damaged by the pathogen (Figure 1) and needs to respond to overcome the disease. In addition, it is essential to know the level of infection of the samples at the time of sample collection rather than the day post-infection, so that sample collection will be carried out on the same plant population on which the evolution of the disease is being tested. The results indicate a relationship between the damage observed in the population and the response associated with the infection. The inoculated samples collected on day 3 had slight damage (Table 1), which could explain the minor differences in gene expression between inoculated and non-inoculated samples (Table 2) compared to the following days. Furthermore, it must be taken into account that if a tissue is very damaged, the gene expression response to the pathogen may not be adequate. This could explain the case of susceptible plants on day 14 post-inoculation (Table 1), whose changes in gene expression between inoculated and non-inoculated samples did not show differences in *CpDEF* in both tissues (Figure 3).

A plant’s defense against pathogens is a process regulated by a complex network of gene families that are also involved in other processes, especially in the case of hormones. The hormonal and enzymatic responses are closely related, as noted in melon challenged by different pathogens, where chitinase and peroxidase activity increased with ethylene and methyl jasmonic gas treatment [35]. Many of them share pathways and functions, such as *CpPAL* and *CpLPOX* in lignin formation [36], so elucidating the specific basis of resistance requires a broad study of all agents involved. 

## 4. Materials and Methods

### 4.1. Materials, Test Conditions, and Inoculation

Two *Cucurbita* genotypes were used, tolerant *Cucurbita moschata* (cv. M63, Rijk Zwaan 64-063) [37] and susceptible *Cucurbita pepo* subsp. *pepo* (cv. MUCU-16, IFAPA-La Mojonera seed collection). Before transplanting, seeds were disinfected by immersing them in a sodium hypochlorite (35 g L^−1^ active chloride) solution for 20 min, rinsing with tap water, and incubating at 28 °C in the dark (wrapped in wet filter paper) until the root reached 3–5 cm in length. Only germinated seeds were potted, so it was necessary to arrange sowing and plant development tests beforehand to estimate the date for both species in order to inoculate them simultaneously, because resistance is closely linked to plant development [38]. Both genotypes had germination rates higher than 85% after one week and reached the three true leaves required for inoculation 8–10 days after germination. The substrate used for the experiments was autoclaved (121 °C, 30 min) vermiculite at field capacity with a standard nutrient solution (1.5 dS m^−1^). 

*Phytophthora capsici* isolate (MI0211) was recovered from infected zucchini in a greenhouse [2] and subsequently inoculated on *Cucurbita pepo* and obtained again from dead plant roots. *P. capsici* was inoculated by irrigation through a 50 mL suspension containing 2.4 × 10^4^ CFU mL^−1^ propagules at 2 cm depth next to the plant’s basal stem, when the plants reached the development of 2–3 true leaves. This solution was obtained by grinding several colonies fully covering the dish surface (10 days) of the isolate previously grown in PDA in nutrient solution. The rate was one plate per 400 mL of final suspension. The inoculum consisted mainly of mycelia, and the concentration was calculated a posteriori using the PDA dilution plate method [39]. The controls were plants not inoculated but watered with a homogenized blend of non-colonized PDA and nutrient solution. Fertigation with nutrient solution was applied until the end of the test according to the plant’s needs, in an effort to maintain the substrate close to saturation [2]. Tests were performed in a growth chamber with a 14 h photoperiod (>12,000 lux) at 20–33 °C (20–30 °C inside the pot) and 45–75% relative humidity. Temperature and humidity were measured using a HOBO data logger (Onset Computer Co., Bourne, MA, USA). 

Plants were distributed randomly in the chamber in 16 trays, each one containing two 1 L pots per genotype (2 plants/pot); 8 trays were inoculated and 8 were non-inoculated. A total of 64 plants per genotype, 32 inoculated and 32 non-inoculated, were used. Replicates for the phenotypic analysis (Table 1) were decreased by 8 per day in the qPCR sample collection, and there were always 8 plants for qPCR analysis. 

### 4.2. Sample Collection and qPCR Conditions

Crown and root samples were collected on days 0, 3, 10, and 14 post-inoculation, and the disease evolution was observed by means of a disease severity index (DSI) from 0 to 4: 0 = no symptoms; 1 = leaf decay/crown scar; 2 = soaked or girdling crown; 3 = wilt and chlorosis; 4 = death (Figure 1) [28]. Crown samples were collected from the last 2 cm of the basal stem and root samples contained the roots closest to the stem, including main and secondary roots. Each plant sample was randomly selected from 4 different trays for the same collection time. The entire plant was removed from the pot and immersed in sterile distilled water to remove any remaining vermiculite. Then, both tissues were wrapped in tinfoil, frozen with liquid nitrogen, and preserved at −80 °C until RNA extraction [10]. 

Samples were homogenized in a mortar with liquid nitrogen and the RNA was extracted using TRIzol reagent (Ambion, Thermo Fisher Scientific, Waltham, MA, USA). Each RNA sample came from 8 different plants, and quality and concentration were checked by electrophoretic gel and NanoDrop2000c (Thermo Scientific, Waltham, MA, USA). DNase I (Invitrogen, Waltham, MA, USA) was applied to remove remaining genomic material, and it was transformed into cDNA using a high-capacity cDNA reverse transcription kit (Applied Biosystems, Waltham, MA, USA). The cDNA obtained was used for qPCR analysis with the PowerUP SYBR green master mix kit (Applied Biosystems, Waltham, MA, USA) in a LightCycler^®^ 96 (Roche Diagnostic, Basel, Switzerland), with three replicates. All enzymes and reagents mentioned were used according to the manufacturer’s instructions.

The primers (Sigma-Aldrich, St. Louis, MO, USA) (Table 8) were designed using Primer3 plus software [40]. Multiple sequence alignment for the primers and both genotypes was done with the EMBL-EBI Clustal omega tool [41] to check that the qPCR would produce the same amplicon in *C. pepo* subsp. *pepo* and *C. moschata*. The expected size of each amplicon was verified by agarose gel electrophoresis (1.5%). For the gene target selection, due to the wide variety of enzymes within each family of the selected pathogenic-related proteins and hormones, we based it on those that presented more homology with other authors’ gene-expression studies in cucurbits and soil-borne pathogenic fungi [10,11,42,43]. 

The qPCR conditions were as follows: 50 °C for 2 min, 95 °C for 10 min, 40 × (95 °C for 15 s, 55 °C for 15 min, 60 °C for 1 s); melting (95 °C for 15 s, 60 °C for 1 min, 95 °C for 1 s). Gene expression was relativized with UFP and EF-1a reference genes [29] following data processing with efficiency correction, multiple reference gene normalization, and the use of error propagation rules [44,45]. Similar melting peak curves for the same gene for the different samples and low reference genes deviations were checked with LightCycler^®^ 96 software v. 1.1 (Roche Diagnostic, Basel, Switzerland). 

For statistical analysis, different factors were considered: genotype (levels: M63 and MUCU-16), tissue (levels: crown and root), inoculum (levels: inoculated and non-inoculated), and days post-inoculation (levels: 3, 10, and 14 DPI). Data are expressed as normalized relative quantity (NRQ; Figure 2), and when comparing two factors, the results are shown as 10-fold relative change (log_10_(NRQ_a_/NRQ_b_); Figure 3, Figure 4 and Figure 5). Comparisons were always made between two levels, pooling the largest possible number of samples from different factors. Data pooling is described in Table 2, Table 3, Table 4, Table 5, Table 6 and Table 7. Data analysis of significant differences (*p* < 0.05) in NRQ (with different log_10_ transformations) was carried out by Student’s *t*-test following homoscedasticity and normal distribution testing. Kruskal–Wallis test was used for the non-parametric DSI test. Statistix v.9.0 (Tallahassee, FL, USA) was used for the analyses.

## 5. Conclusions

To our knowledge, this is the first gene expression study of crown rot caused by *Phytophthora capsici* in *Cucurbita* spp. The results indicate that there were gene expression differences between inoculated and non-inoculated samples, genotypes (*C. moschata* and *C. pepo* subsp. *pepo*), tissues (crown and root), and days post-inoculation.

The data show higher expression changes between inoculated and non-inoculated samples on days 10–14 than on day 3, suggesting a correlation between the DSI observed and plant response. There was overexpression in inoculated samples for *CpDEF, CpLPOX*, and *CpACO* genes. Some differentially expressed genes between crown and root were found. *CpChiIV* was more expressed in crown than in root for both genotypes, while for MUCU-16, *CpACO* and *CpLPOX* expression were higher in root and crown, respectively. There was an important disease influence on the tissue pattern expression of *CpDEF*, since defensin overexpression in inoculated samples was higher in the crown than in the roots. The most overexpressed gene was *CpDEF* in the tolerant species, which also showed significant differences with respect to the susceptible species, especially in the crown. 

These results indicate that *CpPAL* downregulation could be associated with susceptibility, while the maintenance of *CpDEF* overexpression could be related to tolerance. In addition, defensin overexpression could be used as a marker of biotic stress, because it was expressed in larger quantities, was upregulated at early disease stages, and is present in many crops.

## Figures and Tables

**Table 1:** Disease development of inoculated plants.

Days Post Inoculation	Species	Number of Evaluated Plants ^2^	DSI ^1^	Plant Showing Symptoms (%)
3	MUCU-16 M63	38	0.05 ± 0.32	2.6
		32	0	0
7	MUCU-16 M63	30	0.13 ± 0.73	3.3
		24	0	0
10	MUCU-16 M63	30	0.47 ± 0.94	26.7
		24	0.08 ± 0.28	8.3
14	MUCU-16 M63	22	1.50 ± 1.68	50.0
		16	0.50 ± 1.02	21.4
19	MUCU-16 M63	14	2.14 ± 1.99	57.1
		8	1.00 ± 1.85	25.0

^1^ Mean of disease severity index (DSI): 0 = no symptoms; 1 = leaf decay/crown scar; 2 = soaked/girdling crown; 3 = wilt/chlorosis; 4 = death with standard deviation [28]. Significative differences in DSI were observed between genotypes (*p* = 0.0012, Kruskal–Wallis test) pooling all days (*n* = 373). ^2^ Number of plants decreased by 8 after each qPCR sample collection day (3, 10, 14).

**Table 2:** Gene expression (NRQ) pair comparison between inoculated and non-inoculated samples.

Pooled Data				*p*-Value ^3^				
**DPI**	**Genotypes**	**Tissue**	***N* ^1^**	***CpACO***	***CpPAL***	***CpLPOX***	***CpChiIV***	***CpDEF***
all ^2^	MUC + M63	crown + root	24	-	-	-	-	0.000
all	M63	crown + root	12	-	-	0.007	-	0.000
all	MUC	crown + root	12	-	-	-	-	-
all	MUC + M63	crown	12	-	-	-	-	0.013
all	MUC + M63	root	12	0.001	-	-	-	0.000
3	MUC + M63	crown + root	8	-	-	-	-	-
10	MUC + M63	crown + root	8	-	-	-	-	0.002
14	MUC + M63	crown + root	8	0.008	0.043	-	-	-
10, 14	MUC + M63	crown + root	16	0.028	-	0.045	-	0.000

^1^ Number of samples compared per gen. ^2^ Samples from days 3, 10, and 14 post-inoculation. ^3^ Only *p* < 0.05 indicated, obtained with Student’s *t*-test analysis. DPI, days post-inoculation; *CpACO*, 1-aminocyclopropane-1-carboxylate oxidase; *CpPAL*, phenylalanine ammonia-lyase; *CpLPOX*, lignin-forming peroxidase; *CpChiIV*, endochitinase EP3; *CpDEF*, defensin.

**Table 3:** Gene expression (NRQ) pair comparison between crown and root samples.

Pooled Data				*p*-Value ^3^				
**DPI**	**Genotypes**	**Treatment**	***N* ^1^**	***CpACO***	***CpPAL***	***CpLPOX***	***CpChiIV***	***CpDEF***
all ^2^	MUC + M63	inoc. + non-inoc.	28	-	-	-	0.011	-
all	MUC + M63	non-inoculated	16	-	-	-	0.036	-
all	MUC + M63	inoculated	12	-	-	-	-	-
all	M63	inoc. + non-inoc.	14	-	-	-	0.024	-
all	MUC	inoc. + non-inoc.	14	0.002	-	0.042	0.029	-

^1^ Number of samples compared per gen. ^2^ Samples from days 0, 3, 10, and 14 post-inoculation. ^3^ Only *p* < 0.05 indicated, obtained with Student’s *t*-test analysis. DPI, days post-inoculation; *CpACO*, 1-aminocyclopropane-1-carboxylate oxidase; *CpPAL*, phenylalanine ammonia-lyase; *CpLPOX*, lignin-forming peroxidase; *CpChiIV*, endochitinase EP3; *CpDEF*, defensin.

**Table 4:** Gene expression (NRQ) pair comparison between M63 and MUCU-16 samples.

Pooled Data				*p*-Value ^3^				
**DPI**	**Tissues**	**Treatment**	***N* ^1^**	***CpACO***	***CpPAL***	***CpLPOX***	***CpChiIV***	***CpDEF***
all ^2^	crown + root	inoc. + non-inoc.	28	0.004	0.035	0.000	0.001	-
all	crown + root	non-inoculated	16	0.023	0.002	0.002	0.003	0.030
all	crown + root	inoculated	14	0.040	-	0.027	-	0.023
all	crown	inoc. + non-inoc.	14	0.002	-	0.000	0.047	-
all	root	inoc. + non-inoc.	14	-	0.025	-	0.002	-

^1^ Number of samples compared per gen. ^2^ Samples from days 0, 3, 10, and 14 post-inoculation. ^3^ Only *p* < 0.05 indicated, obtained with Student’s *t*-test analysis. DPI, days post-inoculation; *CpACO*, 1-aminocyclopropane-1-carboxylate oxidase; *CpPAL*, phenylalanine ammonia-lyase; *CpLPOX*, lignin-forming peroxidase; *CpChiIV*, endochitinase EP3; *CpDEF*, defensin.

**Table 5:** Gene expression (Log_10_(NRQ_inoculated_/NRQ_non-inoculated_)) pair comparison between MUCU-16 and M63.

Pooled Data			*p*-Value ^3^				
**DPI**	**Tissues**	***N* ^1^**	***CpACO***	***CpPAL***	***CpLPOX***	***CpChiIV***	***CpDEF***
all ^2^	crown + root	12	-	0.021	-	-	0.012
all	crown	6	-	0.017	-	-	-
all	root	6	-	-	-	-	-
10, 14	crown + root	8	-	0.021	-	-	0.043

^1^ Number of samples compared per gen. ^2^ Samples from days 3, 10, and 14 post-inoculation. ^3^ Only *p* < 0.05 indicated, obtained with Student’s *t*-test analysis after log_10_(NRQ_inoculated_/NRQ_non-inoculated_) data transformation. DPI, days post-inoculation; *CpACO*, 1-aminocyclopropane-1-carboxylate oxidase; *CpPAL*, phenylalanine ammonia-lyase; *CpLPOX*, lignin-forming peroxidase; *CpChiIV*, endochitinase EP3; *CpDEF*, defensin.2.4. Differentially expressed genes between root and crown tissues.

**Table 6:** Gene expression (log_10_(NRQ_crown_/NRQ_root_)) pair comparison between inoculated and non-inoculated samples.

Pooled Data			*p*-Value ^3^				
**DPI**	**Genotypes**	***N* ^1^**	***CpACO***	***CpPAL***	***CpLPOX***	***CpChiIV***	***CpDEF***
all ^2^	MUCU-16 + M63	12	-	-	-	-	-
10, 14	MUCU-16 + M63	8	-	-	-	-	0.032
10, 14	MUCU-16	4	-	-	-	0.037	0.047
10, 14	M63	4	-	-	-	-	0.021

^1^ Number of samples compared per gen. ^2^ Samples from days 3, 10, and 14 post-inoculation. ^3^ Only *p* < 0.05 indicated, obtained with Student’s *t*-test analysis after log_10_(NRQ_crown_/NRQ_root_) data transformation. DPI, days post-inoculation; *CpACO*, 1-aminocyclopropane-1-carboxylate oxidase; *CpPAL*, phenylalanine ammonia-lyase; *CpLPOX*, lignin-forming peroxidase; *CpChiIV*, endochitinase EP3; *CpDEF*, defensin.

**Table 7:** Gene expression ((Log_10_(NRQ_M63_/NRQ_MUCU-16_)) pair comparison between inoculated and non-inoculated samples.

Pooled Data			*p*-Value ^3^				
**DPI**	**Tissue**	***N* ^1^**	***CpACO***	***CpPAL***	***CpLPOX***	***CpChiIV***	***CpDEF***
all ^2^	crown + root	12	-	-	-	-	0.000
all	crown	6	-	-	-	0.035	0.000
all	root	6	-	-	-	-	0.024
10, 14	crown + root	8	-	0.013	-	-	0.035

^1^ Number of samples compared per gen. ^2^ Samples from days 3, 10 and 14, post-inoculation. ^3^ Only *p* < 0.05 indicated, obtained with Student’s *t*-test analysis after log_10_(NRQ_M63_/NRQ_MUCU-16_) data transformation. DPI, days post-inoculation; *CpACO*, 1-aminocyclopropane-1-carboxylate oxidase; *CpPAL*, phenylalanine ammonia-lyase; *CpLPOX*, lignin-forming peroxidase; *CpChiIV*, endochitinase EP3; *CpDEF*, defensin.

**Table 8:** Primers and *C. pepo* and *C. moschata* targeted transcripts for rt-qPCR analysis.

Code ^1^	Target mRNA	Homology (%)	Primers (5′-3′)		Size (pb)
*CpACO*	XM_023673456.1 XM_023075072.1	98.2	AGGTTTAAGGAGGCTGTGGC	AACGTGCTTTCCCAGTCCAT	80
*CpChiIV*	XM_023664295.1 XM_023071306.1	98.3	GGAGGAGTTCTTCAACGGCA	ACGATTGGAGGGCTTCAAGG	98
*CpDEF*	XM_023694257.1 XM_023084199.1	87.5 ^2^	CAACTTCAGGGGGCTATGCT	TGCAAGCGCCATCGTTAAAC	80
*CpPAL*	XM_023688417.1 XM_023070093.1	92.6	CCTTCAAATCTCTCTGCAAG	AGATATTGAAGCTCAGAGC	98
*CpLPOX*	XM_023674350.1 XM_023077519.1	97.0	CTGTCGGTGGTCCATCTTGG	TGAAAGTGTGGAAGCTCGCT	94

^1^ Primer alignment of ubiquitin fusion protein (UFP) and elongation factor-1a (EF-1a) reference genes [29] also checked for both species. ^2^ Protein motif alignment between *C. moschata* and *C. pepo* was 100% homologous (Appendix A, Figure A1). *CpACO*, 1-aminocyclopropane-1-carboxylate oxidase; *CpChiIV*, endochitinase EP3; *CpDEF*, defensin; *CpPAL*, phenylalanine ammonia-lyase; *CpLPOX*, lignin-forming peroxidase.

## Data Availability

Date is contained within the article
